# Co-Loading of Ascorbic Acid and Tocopherol in Eudragit-Nutriosomes to Counteract Intestinal Oxidative Stress

**DOI:** 10.3390/pharmaceutics11010013

**Published:** 2019-01-04

**Authors:** Maryam Rezvani, Maria Letizia Manca, Carla Caddeo, Elvira Escribano-Ferrer, Claudia Carbone, José Esteban Peris, Iris Usach, Octavio Diez-Sales, Anna Maria Fadda, Maria Manconi

**Affiliations:** 1 Department of Scienze della Vita e dell’Ambiente, University of Cagliari, via Ospedale 72, 09124 Cagliari, Italy; maryrezvani.unica@gmail.com (M.R.); caddeoc@unica.it (C.C.); mfadda@unica.it (A.M.F.); manconi@unica.it (M.M.); 2 Department of Food Science and Technology, Faculty of Agriculture, University of Tabriz, 51666-16471 Tabriz, Iran; 3 Department of Pharmacy and Pharmaceutical Technology and Physical-Chemistry, Faculty of Pharmacy and Food Science, Institut of Nanoscience and Nanotechnology (IN2UB), University of Barcelona, Av. Joan XXIII s/n, 08028 Barcelona, Spain; eescribano@ub.edu; 4 Department of Scienze del Farmaco, University of Catania, Viale A. Doria 6, 95125 Catania, Italy; carbone.claudia@gmail.com; 5 Department of Pharmacy, Pharmaceutical Technology and Parasitology, University of Valencia, Avda Vicente Andrés Estellés s/n, 46100 Burjassot, Valencia, Spain; Jose.E.Peris@uv.es (J.E.P.); Iris.Usach@uv.es (I.U.); octavio.diez@uv.es (O.D.-S.); 6 Instituto de Reconocimiento Molecular y Desarrollo Tecnológico, Centro Mixto Universidad Politécnica de Valencia, Universidad de Valencia, Avda Vicente Andrés Estellés s/n, 46100 Burjassot, Valencia, Spain

**Keywords:** Eudragit, Nutriose, phospholipid vesicles, antioxidant, intestinal wound healing

## Abstract

The present study aimed at developing a new vesicular formulation capable of promoting the protective effect of ascorbic acid and tocopherol against intestinal oxidative stress damage, and their efficacy in intestinal wound healing upon oral administration. A pH-dependent copolymer (Eudragit^®^ L100), a water-soluble prebiotic fibre (Nutriose^®^ FM06), a phospholipid mixture (Lipoid S75), and two natural antioxidants (ascorbic acid and tocopherol) were combined to fabricate eudragit-nutriosomes by a simple, solvent-free procedure. The vesicles were spherical and oligolamellar, with some multicompartment structures in Eudragit-nutriosomes, small in size (~100 nm), with highly negative zeta potential. The effect of Eudragit^®^ and Nutriose^®^ on the stability on storage and in simulated gastrointestinal fluids were confirmed by the Turbiscan^®^ technology and in vitro studies, respectively. Eudragit-nutriosomes exhibited a protective effect against H_2_O_2_-induced oxidative stress, and a proliferative effect in Caco-2 cells, as they provided the closure of the scratched area after 96 h of incubation.

## 1. Introduction

Alpha-tocopherol is a potent antioxidant and the most bioactive form of vitamin E with extended use as a component of pharmaceutical, cosmetic and functional food formulations. This lipid-soluble antioxidant is abundantly found in many natural sources, such as nuts, seeds and vegetable oils, and usually formulated with hydro-soluble antioxidant ascorbic acid (vitamin C) to enhance antioxidant effects [1,2]. Since the human body is not able to store a large amount of ascorbic acid, it has to be consumed every day to achieve its bio-functional effect. Fruits, vegetables, and their extracts are the best sources of ascorbic acid [3]. Despite the beneficial effects of these antioxidants against inflammation, oxidative stress, and chronic gastrointestinal disorders via quenching of oxidizing free radicals [4,5,6,7,8], their practical applications are challenging due to their rapid degradation and sensitivity to environmental factors, e.g., oxygen, heat, and light. Nanoencapsulation has been proposed as an efficient strategy to overcome these obstacles [9]. Liposomes are the most well-known and studied carrier systems in the biomedical field [10]. Recently, we have introduced nutriosomes, a novel type of phospholipid nanocarrier containing a prebiotic soluble dextrin, Nutriose^®^, and a natural antioxidant, curcumin, which have shown promising efficacy against intestinal damage in vivo [11]. Nutriose^®^ is an indigestible dextrin fibre from maize that, like other prebiotics, stimulates the growth and/or activity of the gut microbiome, thus reducing the risk for intestinal disorders and improving health.

Targeted drug delivery is one of the important research fields in modern pharmaceutical and medical sciences. Methacrylate copolymers (Eudragit^®^) are an appropriate choice for the development of intestinal targeted drug delivery systems; they are pH-responsive enteric polymers that can be exploited to target different regions of the intestine for the treatment of many diseases such as inflammatory bowel disease (IBD) and colorectal cancer [12].

In this study, the synergistic effects of alpha-tocopherol, ascorbic acid, Nutriose^®^ FM06, and Eudragit^®^ L100 combined in a novel nanocarrier system against intestinal oxidative stress were investigated. Dynamic light scattering, cryo-TEM and Turbiscan^®^ technology were employed to evaluate the colloidal features and the stability of the nanovesicles. Moreover, their behavior in simulated gastrointestinal fluids and their protective effect against oxidative stress were assessed in vitro, along with the efficacy in intestinal wound healing.

## 2. Materials and Methods

### 2.1. Materials

Lipoid S75 containing phosphatidylcholine (~70%), phosphatidylethanolamine (~10%), lysophosphatidylcholine (~3%), triglycerides (~3%), fatty acids (~0.5%) and tocopherol (0.1–0.2%), was purchased from Lipoid GmbH (Ludwigshafen, Germany). Nutriose FM06^®^, a soluble dextrin from maize, was kindly provided by Roquette (Lestrem, France). Eudragit^®^ L100 (EU) with molecular weight about 125,000 g/mol was kindly provided by Evonik Industries AG (Darmstadt, Germany). Ascorbic acid, 2,2-diphenyl-1-picrylhydrazyl (DPPH), ethanol, and other reagents of analytical grade were purchased from Sigma-Aldrich (Milan, Italy). Alpha-tocopherol acetate was purchased from Galeno (Carmignano, Potenza, Italy). Cell medium, foetal bovine serum, penicillin, and streptomycin were purchased from Life Technologies Europe (Monza, Italy).

### 2.2. Sample Preparation

To prepare nutriosomes and EU-nutriosomes, 60 mg/mL of S75 phospholipid, 10 mg/mL of ascorbic acid, 5 mg/mL of tocopherol, 25 mg/mL of Nutriose^®^ FM06 and 0.5 mg/mL Eudragit^®^ L100 when appropriate, were left swelling for 1 h with 2 mL of bi-distilled water, and sonicated (3 s on and 2 s off, 15 cycles; 13 micron of probe amplitude) with a high intensity ultrasonic disintegrator (Soniprep 150, MSE Crowley, London, UK). After the complete cooling of the sample, the sonication was repeated (5 cycles, 3 s on and 2 s off). Liposomes without Nutriose^®^ or Eudragit^®^ were prepared by the same procedure and used as a reference.

### 2.3. Vesicle Characterization

Cryo-TEM analyses were performed to evaluate vesicle formation and morphology as previously reported [13].

M3-PALS (Mixed Mode Measurement-Phase Analysis Light Scattering) technique and Photon Correlation Spectroscopy (PCS) were used to evaluate vesicle surface charge, average diameter and polydispersity index (PI) respectively, by means of a Zetasizer nano-ZS (Malvern Instruments, Worcestershire, UK). Samples (100 μL) were diluted with water (10 mL) prior to analysis.

Purification of samples was performed by dialysis. For each sample, 2 mL was taken and loaded into dialysis tubing (Spectra/Por^®^ membranes, 12–14 kDa MW cut-off, 3 nm pore size; Spectrum Laboratories Inc., DG Breda, The Netherlands) and maintained in water (3 L, refreshed every hour) for 2 h to ensure the removal of the non-incorporated compounds. The dispersions before and after dialysis were diluted with methanol (1:1000) to disrupt the vesicles and dissolve the components. The amounts of ascorbic acid and tocopherol were quantified by HPLC with UV detection at 285 nm and 290 nm, respectively. In the case of ascorbic acid, a Waters Spherisorb S5 ODS2 column (4.6 × 250 mm) was used, and the mobile phase consisted of a mixture of methanol and sodium phosphate monobasic buffer (25 mM, pH 4.6) (5:95, *v*/*v*). The injection volume was 25 µL, and the flow rate was 0.5 mL/min. In the case of tocopherol, a Waters Nova-Pack C18 analytical column was used, the mobile phase consisted of a mixture of acetonitrile and methanol (99:1 *v*/*v*), delivered at a flow rate of 1 mL/min, and the injected volume was 25 µL.

The entrapment efficiency (EE) was calculated as the percentage of the amount of ascorbic acid and tocopherol recovered in dialyzed dispersions versus the amount found before dialysis.

### 2.4. Stability of Vesicle Dispersions

The stability of the formulations was evaluated by standard long-term stability tests, i.e., analyzing vesicle mean size and polydispersity over 2 months at room temperature, and by using the optical analyzer Turbiscan^®^ Ageing Station (Formulaction, l’Union, France), which exploits the Static Multiple Light Scattering for the analysis of dispersions. 20 mL of each sample was placed in a cylindrical glass cell of the ageing station at 25 °C for 30 days. The entire height of the sample cell was scanned at programmed times, and the detected destabilization phenomena were reported in a graph showing the variation of transmission (ΔT, in ordinate) and the height of the cell (in abscissa). The Turbiscan Stability Index (TSI) was also measured to evaluate the stability behaviour of the formulations.

### 2.5. Vesicle Behaviour in Gastrointestinal Fluids

Two different media (at pH 1.2 and at pH 7.0) both containing sodium chloride to increase the ionic strength, were used to simulate the gastrointestinal environment. Samples were diluted (1:100 *v*:*v*, at 37 °C) with the media, and variations in terms of vesicle average diameter, polydispersity index and zeta potential were measured immediately after dilution and after incubation for 2 h or 6 h at pH 1.2 or 7.0, respectively.

### 2.6. Biocompatibility of the Vesicles in Caco-2 Cells

The biocompatibility of ascorbic acid and tocopherol in aqueous solution or co-loaded in liposomes, nutriosomes, and EU-nutriosomes was evaluated using Caco-2 cells. The cells were cultured in Dulbecco’s Modified Eagle Medium containing high glucose and l-glutamine, and supplemented with penicillin-streptomycin, fungizone, and foetal bovine serum (FBS, 10%) in 5% CO_2_ at 37 °C to allow exponential growth.

7.5 × 10^3^ cells/well were seeded for 24 h into 96-well plates and then treated for 48 h with the ascorbic acid and tocopherol in aqueous solution or co-loaded in liposomes, nutriosomes and EU-nutriosomes at different dilutions corresponding to 0.01, 0.1, 1, and 10 μg/mL ascorbic acid and 0.005, 0.05, 0.5, and 5 μg/mL tocopherol. The MTT [3(4,5-dimethylthiazolyl-2)-2,5-diphenyltetrazolium bromide] colorimetric assay was used to measure the cell viability. 100 µL of MTT reagent (0.5 mg/mL in PBS) was added to each well, and after 3 h, DMSO was used to dissolve the formed formazan crystals, which were quantified spectrophotometrically at 570 nm with a microplate reader (Multiskan EX, Thermo Fisher Scientific Inc., Waltham, MA, USA) [14].

### 2.7. Antioxidant Activity of the Vesicles

The DPPH assay was used to measure the in vitro antioxidant activity of ascorbic acid and tocopherol in aqueous solution and co-loaded in liposomes, nutriosomes and EU-nutriosomes. 10 μL of each sample was dissolved in 1990 μL of DPPH methanolic solution (40 μg/mL), incubated at room temperature in the dark for 30 min, and the absorbance was read at *λ* = 517 nm. The antioxidant activity (*AA*%) was calculated according to the following equation:

*AA*% = [(*A* − *B*)/*A*] × 100 (1)

where *A* and *B* were the absorbance of DPPH and sample solutions, respectively.

Additionally, the antioxidant activity of ascorbic acid and tocopherol in aqueous solution or co-loaded in the vesicles was assessed in cell culture [13]. Caco-2 cells were seeded into 96-well plates at a density of 7.5 × 10^3^ cells/well. After 24 h of incubation, the cells were exposed to hydrogen peroxide (1:40,000) in the presence or absence of the ascorbic acid and tocopherol aqueous solution or vesicle formulations (final concentration of ascorbic acid and tocopherol: 1 and 0.5 μg/mL, respectively). The cells exposed to hydrogen peroxide only were used as a positive control. The MTT test was used to measure cell viability, which was expressed as the percentage of untreated cells (100% viability).

### 2.8. Intestinal Wound Healing Activity of the Vesicles

The scratch assay was performed to evaluate the ability of ascorbic acid and tocopherol to stimulate the proliferation and migration of Caco-2 cell. The experiment was carried out in 6-well plates; cells were cultured until confluence was reached. At day 0, a linear scratch was generated using a sterile pipette tip, and each well was gently washed to remove the scattered fragments of the cells. Once the wound was generated, the cells were treated with ascorbic acid and tocopherol in aqueous solution or loaded in vesicles, and incubated for 48 h and 96 h. Untreated cells were used as a control. At the end of the experiments, the cells were observed under a light microscope using a 10× objective.

### 2.9. Statistical Analysis of Data

Results are expressed as means ± standard deviations. Multiple comparison of means was evaluated using ANOVA, while differences between groups were studied by Tukey’s test. The differences were considered statistically significant for *p* < 0.05.

## 3. Results

### 3.1. Vesicle Preparation and Characterization

**Figure 1:**
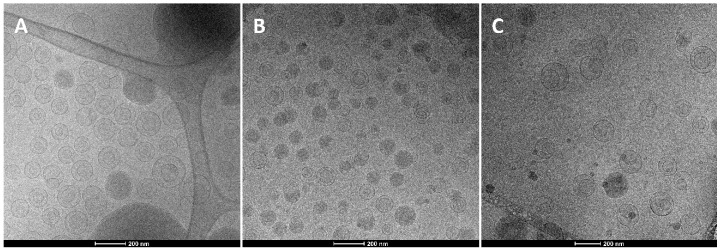
Representative cryo-TEM images of ascorbic acid and tocopherol co-loaded liposomes (**A**), nutriosomes (**B**), and EU-nutriosomes (**C**).

Nutriosomes, EU-nutriosomes, and liposomes were produced by a simple, organic solvent-free procedure involving the hydration and sonication of the formulation components in aqueous dispersion. Cryo-TEM was employed to confirm the formation of the vesicles and evaluate their morphology (Figure 1). 

Liposomes were spherical in shape and mainly uni- or bi-lamellar vesicles; the addition of Nutriose^®^ in nutriosomes did not modify significantly the vesicle morphology, while the co-presence of Nutriose^®^ and Eudragit^®^ led to the formation of oligolamellar and multicompartment structures [11].

The small diameters and low PI values indicated the high efficiency of the preparation technique for the three nano-systems. The addition of Nutriose FM06^®^ and Eudragit^®^ L100 to the liposomal formulation led to a slight increase in size and a decrease in the PI to less than 0.1. All the formulations possessed highly negative zeta potential, which ensured appropriate stability. The entrapment efficiency of tocopherol was low (~10%), probably due to the fact that it has some water solubility (~0.5 mg/mL), while the values for ascorbic acid were unexpectedly high (>70%). More efforts will be devoted toward finding new manufacturing procedures or components of the formulations, so as to improve the entrapment efficiency of tocopherol, Table 1.

### 3.2. Vesicle Stability

The stability of the formulations was evaluated by standard long-term stability tests, i.e., analyzing vesicle mean size and polydispersity over two months at room temperature. No signs of physical alteration were observed, as confirmed by the fact that the vesicle size was basically unchanged (±10%; *p* < 0.05) over the storage period.

**Figure 2:**
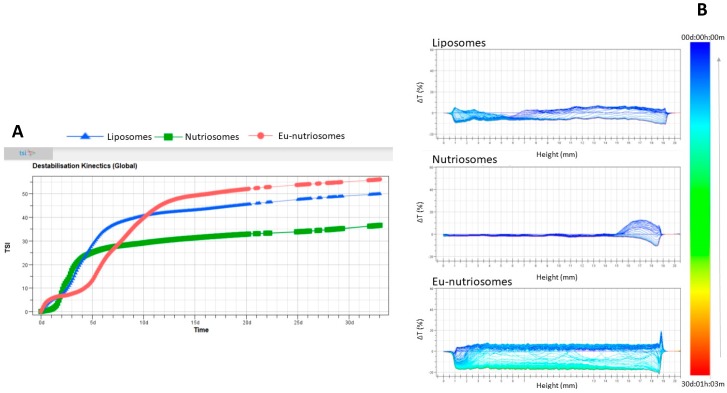
Evolution of Turbiscan^®^ Stability Index (TSI) for ascorbic acid and tocopherol co-loaded liposomes, nutriosomes and EU-nutriosomes over 30 days at 25 °C (**A**). Turbiscan^®^ ΔT profiles for ascorbic acid and tocopherol co-loaded liposomes, nutriosomes and EU-nutriosomes over 30 days at 25 °C (**B**).

The physical stability, in terms of particle migration and/or aggregation phenomena, was evaluated by the Turbiscan^®^ technology. As reported in Figure 2, TSI profiles highlight that the addition of eudragit in the nutriosomal formulation was able to increase the short-term stability of the vesicles, since after one week no significant variation in TSI index was observed in EU-nutriosomes. 

However, after five days EU-nutriosomes underwent a significant instability phenomenon, the intensity of which was greater than in the other formulations. Interestingly, nutriosomes showed a greater long-term stability, as compared to liposomes and EU-nutriosomes, as clearly reported by the lower TSI indexes profile after 30 days of storage. The transmission profiles of the formulations, reported in Figure 2B (ΔT profiles), clearly highlight important differences in the vesicles’ stability, depending on their structure and composition. Liposomes and EU-nutriosomes showed the occurrence of significant instability phenomena (ΔT > 20%) related to vesicle aggregation, without vesicle migration at the top or bottom of the cuvette. On the contrary, nutriosomes showed greater stability, since no vesicle aggregation was shown in the middle of the cuvette (ΔT < 2%), and only a slight migration was observed at the bottom of the cuvette (ΔT ≤ 10%). It has to be pointed out that even when instability phenomena occurred, the reversibility of the separation allowed the re-suspension of the vesicles by gentle shaking.

### 3.3. Vesicle Behaviour in Gastrointestinal Fluids

The stability of the vesicles in simulated gastrointestinal fluids (SGIF) was investigated by incubating the formulations at pH 1.2 and 7.0 in the presence of 0.3 M sodium chloride, at 37 °C, and measuring their size, PI and zeta potential (Table 2).

The acidic medium caused an increase in size for both liposomes and nutriosomes (from ~100 to ~200 nm; Table 1 and Table 2), and after 2 h at pH 1.2, a further increase was observed (to ~260 and 360 nm, respectively). The polydispersity index increased as well, from ~0.10 to ~0.50. On the contrary, in spite of the harsh conditions tested, the EU-nutriosomes preserved their structure almost entirely, as their mean diameter and polydispersity were not significantly affected, as compared to the starting values (from ~110 to ~150 nm; PI from 0.10 to ~0.20). The stability of EU-nutriosomes was clearly due to the presence of Eudragit^®^, which provided gastro-resistance and entero-solubility to the vesicles (Eudragit^®^ dissolves at pH > 6).

After 6 h at pH 7.0, the size and polydispersity index of the three formulations remained basically unchanged (*p* > 0.05). Furthermore, the zeta potential of the vesicular formulations turned to positive values in both simulated medias.

### 3.4. Vesicle Biocompatibility In Vitro

**Figure 3:**
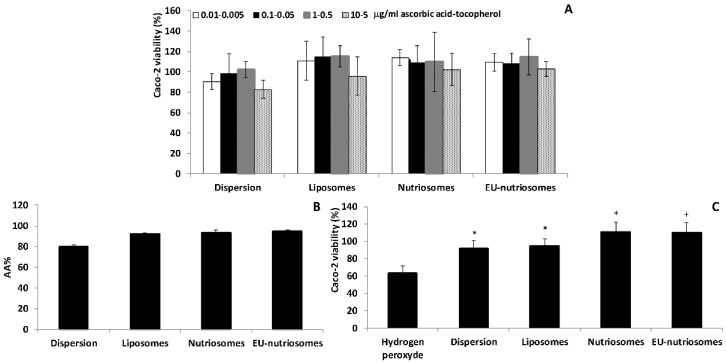
Viability of Caco-2 intestinal cells incubated for 48 h with ascorbic acid and tocopherol in aqueous solution or co-loaded in liposomes, nutriosomes and EU-nutriosomes (**A**). Data are reported as mean values ± standard deviations of cell viability expressed as the percentage of control cells (100% viability). Antioxidant activity (AA%) of ascorbic acid and tocopherol in aqueous solution or co-loaded in liposomes, nutriosomes and EU-nutriosomes, according to the DPPH assay (**B**). Viability of Caco-2 cells exposed to hydrogen peroxide in the absence or presence of ascorbic acid and tocopherol in aqueous solution or co-loaded in liposomes, nutriosomes and EU-nutriosomes (**C**). Data are reported as mean values ± standard deviation of cell viability expressed as the percentage of the negative control (100% viability). *^,+^ symbols indicate statistically different samples (*p* < 0.05) vs. hydrogen peroxide-treated cells.

In order to evaluate the biosafety of the nanovesicles, the most common human intestinal cell model, i.e., Caco-2 cells, was used (Figure 3). The results from the cell viability studies indicated that ascorbic acid and tocopherol co-loaded vesicles were highly biocompatible, as the viability was higher than 100% at all the concentrations tested, and slightly higher than that obtained with the solution. The biocompatibility seemed to be dose-dependent, and decreased as the concentration of the two vitamins increased.

### 3.5. Antioxidant Activity of the Vesicles

The antioxidant activity of ascorbic acid, tocopherol solution and vesicular formulations was first evaluated as a function of the DPPH-radical scavenging ability of the two vitamins (Figure 3B). Liposomes, nutriosomes and EU-nutriosomes showed a similar inhibitory activity (>90%), which was significantly higher than the solution (79%).

Moreover, the ability of the formulations to protect the Caco-2 cells from oxidative stress induced by the exposure to hydrogen peroxide was assessed (Figure 3C). Hydrogen peroxide is a detrimental oxidant for cells, and significantly decreased the cell viability to approximately 60%, with respect to untreated cells (negative control).

The ascorbic acid and tocopherol solution and liposomes showed a cytoprotective effect against hydrogen peroxide, as the cell viability increased up to ~90%. Nutriosomes (with or without Eudragit^®^) not only protected the cells against oxidative stress, but also exerted a proliferative effect providing a 110% cell viability.

### 3.6. Intestinal Wound Healing Activity of the Vesicles

**Figure 4:**
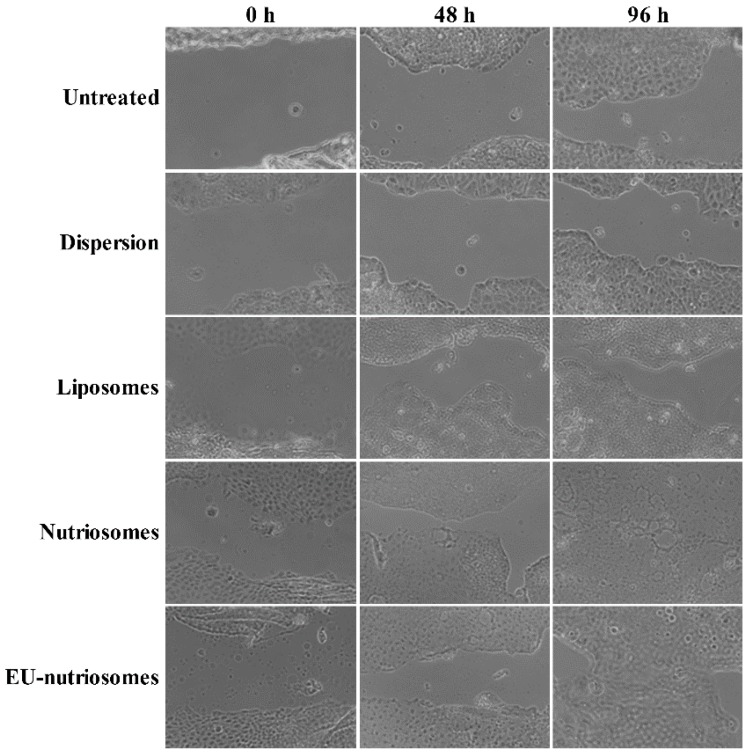
Optical microscopy images of wound closure in intestinal Caco-2 cells as a function of the treatment with ascorbic acid and tocopherol solution or vesicular formulations for 24, 48 and 96 h, in comparison with untreated control cells. Cells were observed under a light microscope using a 10× objective.

The wound healing activity of ascorbic acid, tocopherol solution and liposomes, nutriosomes and EU-nutriosomes was investigated in vitro by monitoring cell expansion on the wound surface leading to wound closure. Neither the solution nor the liposomes were able to effectively stimulate the cell migration and proliferation in the scratched area, so as to achieve a healed closed wound (Figure 4). On the other hand, nutriosomes and EU-nutriosomes accelerated the healing process, and the former provided a complete wound closure after 96 h of incubation, probably due to a synergistic effect of the two antioxidants and Nutriose^®^. In vivo studies will be needed to clarify the mechanisms involved in the efficacy of the tested formulations.

## 4. Discussion

There are several environmental triggers inducing oxidative stress. Acrylamide and trans-fatty acids in processed foods, heavy metals, organic solvents, alcohol, some drugs, pollutants, radiation and smoking can contribute to oxidative stress due to imbalance between pro-oxidants, cellular antioxidants, and excessive generation of reactive oxygen species (ROS). Uncontrolled oxidative stress has a destructive effect leading to chronic inflammatory intestinal disorders, gastrointestinal tract injuries, and carcinogenesis [15,16,17].

The results of the present study showed that the novel vesicular nanocarriers can be proposed as a promising tool to prevent and counteract oxidative stress-related intestinal disorders. For the first time, the co-delivery of tocopherol and ascorbic acid, two natural antioxidants, combined with prebiotic Nutriose FM06^®^ in phospholipid vesicles, namely nutriosomes, was investigated and compared with conventional liposomes. Additionally, Eudragit^®^ L100 polymer was included in the formulation to produce EU-nutriosomes. Eudragit^®^ was expected to allow the delivery of ascorbic acid and tocopherol to the lower part of the gastrointestinal tract, thanks to its pH-dependent behavior. The vesicles were small in size, spherical in shape and mostly uni- and bi-lamellar vesicles. The addition of Eudragit^®^ led to the formation of oligolamellar, multicompartment structures. Their stability was assessed by means of the Turbiscan^®^ technology, which is based on multiple light scattering and provides sensitive identification of destabilization phenomena at an early stage, much faster and more accurate than visual inspection. The TSI profiles obtained in this research indicated that the addition of Eudragit^®^ L100 and Nutriose FM06^®^ in the formulations improved the short-term and long-term stability of the nutriosomal vesicles, respectively, as compared to liposomes.

Since oral delivery systems are exposed to pH and ionic strength variations during their passage through the gastrointestinal tract, the evaluation of the behavior of the liposomal and nutriosomal formulations in SGIF was of great importance. The results suggested that the presence of Eudragit^®^ L100 in the nutriosome formulation led to a greater stability in the simulated gastric environment, which means that EU-nutriosomes can protect the loaded antioxidants from the harsh conditions of the stomach and reach the intestine intact. The biosafety evaluation displayed the absence of cytotoxicity in intestinal cells exposed to ascorbic acid and tocopherol co-loaded nutriosomes and EU-nutriosomes, regardless of the concentration tested.

Antioxidant tests were performed, the results showing that the loading of ascorbic acid and tocopherol in the vesicles, mostly the nutriosomes and EU-nutriosomes, potentiated the DPPH-free radical scavenging activity, as well as the cytoprotective activity against hydrogen peroxide-induced oxidative stress. These effects can be reasonably due to the facilitated cellular internalization of the ascorbic acid and tocopherol by the vesicular carriers, which allowed the antioxidants to exert their radical scavenging activity [18]. Since wound healing has a vital role in the clinical remission of some inflammatory bowel diseases, the effect of liposomal and nutriosomal nanovesicles on wound closure was evaluated. Images showed that nutriosomes and EU-nutriosomes had a superior stimulatory effect on cell expansion on wound surface.

In consideration of these findings, EU-nutriosomes can be proposed as targeted delivery system for counteracting intestinal oxidative stress.

## 5. Conclusions

Ascorbic acid and tocopherol EU-nutriosomal nanovesicles were successfully produced by a simple, environmentally friendly method by combining a prebiotic fibre, an enteric copolymer, and two antioxidants in a phospholipid vesicle system. EU-nutriosomes were demonstrated to have good stability in simulated gastric medium, potent activity against free radicals, and wound healing properties. Therefore, these nanovesicles can represent a promising tool to counteract oxidative stress associated with intestinal injuries.

## Figures and Tables

**Table 1:** Average size, polydispersity index (PI), zeta potential (ZP) and entrapment efficiency (EE) of liposomes, nutriosomes and EU-nutriosomes.

Sample	Size (nm)	PI	ZP (mV)	EE Ascorbic Acid (%)	EE Tocopherol (%)
Liposomes	100 ± 11	0.16	−44 ± 3	82 ± 9	10 ± 8
Nutriosomes	111 ± 8	0.07	−42 ± 3	74 ± 5	12 ± 6
EU-nutriosomes	114 ± 14	0.10	−46 ± 4	76 ± 6	13 ± 9

**Table 2:** Average size, polydispersity index (PI) and zeta potential (ZP) of liposomes, nutriosomes and EU-nutriosomes diluted and incubated at pH 1.2 and pH 7.0, at 37 °C. The measurements were carried out immediately after the dilution (*t*_0_) and after 2 h at pH 1.2 (*t*_2h_) or 6 h at pH 7.0 (*t*_6h_).

Sample	Time	pH 1.2			pH 7.0		
		Size (nm ± SD)	PI	ZP (mV ± SD)	Size (nm ± SD)	PI	ZP (mV ± SD)
Liposomes	*t*_0_	210 ± 22	0.37	+11 ± 2	123 ± 16	0.17	+6 ± 2
	*t*_2/6h_	265 ± 24	0.50	+12 ± 2	141 ± 16	0.23	+7 ± 2
Nutriosomes	*t*_0_	206 ± 40	0.22	+11 ± 3	106 ± 8	0.11	+8 ± 3
	*t*_2/6h_	363 ± 38	0.55	+12 ± 3	112 ± 7	0.09	+7 ± 3
EU-nutriosomes	*t*_0_	153 ± 13	0.17	+6 ± 1	109 ± 9	0.10	+7 ± 2
	*t*_2/6h_	141 ± 16	0.23	+7 ± 1	116 ± 11	0.11	+6 ± 2
